# Immune checkpoint-related serum proteins and genetic variants predict outcomes of localized prostate cancer, a cohort study

**DOI:** 10.1007/s00262-020-02718-1

**Published:** 2020-09-09

**Authors:** Qinchuan Wang, Yuanqing Ye, Hao Yu, Shu-Hong Lin, Huakang Tu, Dong Liang, David W. Chang, Maosheng Huang, Xifeng Wu

**Affiliations:** 1 Department of Surgical Oncology, Affiliated Sir Run Run Shaw Hospital and Department of Epidemiology and Health Statistics School of Public Health, Zhejiang University School of Medicine, Hangzhou, Zhejiang China; 2 Center for Clinical Big Data and Analytics, Bioinformatics and Big Data, The Second Affiliated Hospital and School of Public Health, Zhejiang University School of Medicine, 866 Yuhangtang Rd, Hangzhou, 310058 PR China; 3 Department of Epidemiology, The University of Texas MD Anderson Cancer Center, Houston, TX USA; 4 Division of Cancer Epidemiology and Genetics, National Cancer Institute, Rockville, MD USA; 5 Department of Pharmaceutical Sciences, Texas Southern University, Houston, TX USA

**Keywords:** Serum immune checkpoint, Genetic variations, Localized prostate cancer, Aggressiveness, Biochemical recurrence, Progression free survival

## Abstract

**Background:**

The clinical predictors and biological mechanisms for localized prostate cancer (PCa) outcomes remain mostly unknown. We aim to evaluate the role of serum immune-checkpoint-related (ICK) proteins and genetic variations in predicting outcomes of localized PCa.

**Methods:**

We profiled the serum levels of 14 ICK-related proteins (BTLA, GITR, HVEM, IDO, LAG-3, PD-1, PD-L1, PD-L2, Tim-3, CD28, CD80, 4-1BB, CD27, and CTLA-4) in 190 patients with localized PCa. The genotypes of 97 single nucleotide polymorphisms (SNPs) from 19 ICK-related genes were analyzed in an extended population (*N* = 1762). Meta-data from ArrayExpress and TCGA was employed to validate and to probe functional data. Patients were enrolled and tumor aggressiveness, biochemical recurrence (BCR), and progression information were obtained. Statistical analyses were performed analyzing associations between serum biomarkers, genotypes, mRNA and outcomes.

**Results:**

We showed that serum (s)BTLA and sTIM3 levels were associated with PCa aggressiveness (*P* < 0.05). sCD28, sCD80, sCTLA4, sGITR, sHVEM and sIDO correlated with both BCR and progression risks (all *P* < 0.05). We further identified ICK variants were significantly associated with aggressiveness, BCR and progression. Among them, 4 SNPs located in *CD80* (rs7628626, rs12695388, rs491407, rs6804441) were not only associated with BCR and progression risk, but also correlated with sCD80 level (*P* < 0.01). rs491407 was further validated in an independent cohort. The *CD80* mRNA expression was associated with BCR (HR, 1.85, 95% CI 1.06–3.22, *P* = 0.03) in meta-analysis of validation cohorts.

**Conclusion:**

We highlight the prognostic value of serum ICK-related proteins for predicting aggressiveness, BCR and progression of PCa. The genetic variations and mRNA expression in CD80 could be predictors and potential targets of localized PCa.

**Electronic supplementary material:**

The online version of this article (10.1007/s00262-020-02718-1) contains supplementary material, which is available to authorized users.

## Introduction

Treatment of localized prostate cancer (PCa) is largely dependent on risk stratification based on aggressiveness [1]. Pathological Gleason score (GS) and serum prostate-specific antigen (PSA) are mainly applied in outcome prediction for PCa [2] but remain imperfect in predicting properties of the tumor and biochemical recurrence (BCR), which may lead to over-treatment in patients with indolent cancer [3].

Immunotherapy has shown promise in the battle against PCa. Though Ipilimumab failed in the treatment of metastatic castration-resistant PCa (mCRPC) in a recent clinical trial [4], combinatorial immunotherapy with Immune checkpoint blockade (ICB) and Myeloid-derived suppressor cells (MDSCs) targeted therapy showed efficacy in mCRPC [5]. Overexpression of immune checkpoint (ICK) genes like *HAVCR2* (TIM-3) in T cells could cause dysfunction of PSA-specific CD8+ T cells in PCa [6]. PD-L1 expression is independently associated with BCR in aggressive PCa [7]. These studies implicate the involvement of ICK-related genes during PCa development.

Soluble T cell regulatory proteins (mostly ICK-related proteins) released from immune and tumor cells may affect the efficacy of treatment and outcomes [8]. High soluble (s)PD-L1 is associated with impaired immunity and poor outcomes in aggressive renal cell cancer [9]. However, the association of these markers with PCa outcomes and potential mechanisms is not clear, which requires more studies.

Therefore, we implemented a three-stage study to determine whether soluble ICK-related proteins were associated with PCa outcomes and functionally explored the potential mechanism. First, we evaluated serum levels of 14 ICK-related proteins and their associations with outcomes in 190 patients with localized PCa in the MD Anderson Cancer Center PCa (MDACC-PCa) cohort. Second, the genotype data of 97 single nucleotide polymorphisms (SNPs) from 19 ICK-related genes were obtained in an extended cohort of patients with PCa (*N* = 1762), and the correlation between genotypes and outcomes were further analyzed. Further, we investigated the genotype–phenotype correlation between SNPs and serum ICK-related proteins and used data from ArrayExpress and The Cancer Genome Altas (TCGA) to validate our findings and to derive functional relevance to support our findings.

## Materials and methods

### Study population and data collection

The study was approved by the MDACC Institutional Review Board. Each subject consented to having their clinical data obtained and providing blood samples for research purposes.

This study used data from a previously described cohort of patients with PCa enrolled at MDACC [10, 11]. In brief, the cohort involved non-Hispanic white men with previously untreated PCa. The MDACC-PCa cohort recruited patients who underwent treatment for localized PCa from 2003 to 2013. Clinical data, including diagnosis date, PSA level at diagnosis, biopsy-proven GS, clinical tumor stage, treatment details, tissue pathology, and follow up information were collected from medical records. BCR after local therapy was defined as a single measure of PSA ≥ 0.2 ng/mL after radical prostatectomy [12], or a PSA rise of 2 ng/mL or more above the nadir PSA in patients who received radiotherapy [13]. Progression was defined by either BCR or clinical/radiographic progression of the disease [1, 14]. All pathologic slides from outside institutions were reviewed by a single genitourinary pathologist at MDACC. In all cases, the GS assessed at MDACC was used.

Another independent validation cohort derived from ArrayExpress, which included 132 patients with PCa from Tumour Identity Card Program (CIT), France [15]. All patients were post-prostatectomy, and the criteria for aggressiveness and BCR were the same as for the MDACC-PCa cohort (Table S1).

### Serum ICK-related proteins detection

Samples were tested in duplicate using ProcartaPlex Human Immuno-Oncology Checkpoint Panel (Thermo Fisher, USA) in 96-well plate format to quantify 14 human immune checkpoint markers. Assay was conducted according to protocols provided by the manufacturer using Luminex 200™ instrument and xPONENT® software (Luminex Corp, USA). All inter-assay and intra-assay coefficients of variation (CV) were below 15%.

### Genes of interest in ICK pathway

Based on previous literature, a total of 19 ICK-related genes, which includes most immune checkpoint receptors and ligands, were selected [16]. The chromosome positions of transcriptional start and end points of each gene were obtained from the USCS Genome Browser (https://genome.ucsc.edu/, build version: GRCh37/hg19).

### Genotyping and quality control

All DNA samples were extracted from peripheral whole blood using the QIAamp DNA extraction Kit (QIAGEN). Custom Infinium OncoArray-500 K Beadchip was used to genotype both populations. Assays were run on the iScan system (Illumina, USA). Genotyping data were analyzed and exported using the Genome Studio software (Illumina, USA). All subjects had a call rate > 95%. Genotyping data of 97 SNPs found within 10 kb of upstream and downstream flanking regions for each gene of interest were extracted from the OncoArray dataset and included in the study.

### Functional characterization and immune phenotype association

SNP array (Illumina HumanOmniExpress-12 v1.0) and mRNA expression array (Affymetrix Human Gene 2.0 ST Array) data of 132 patients with PCa from CIT cohort were downloaded from ArrayExpress portal (https://www.ebi.ac.uk/arrayexpress). Bioinformatic analysis was applied to investigate tumor (*N* = 494) and normal tissue (*N* = 52) data of patients with PCa from TCGA databases. Gene level expression based on RNA-seq data (normalized, RSEM level 3) and clinical data for each sample were downloaded directly from the TCGA data and analyzed using Firebrowse API (https://firebrowse.org/). *CD80* mRNA was dichotomized at the median level. Positive Tumor infiltrate lymphocyte (TIL) was defined as TIL percent greater than 0%.

### Statistical analysis

Levels of serum proteins and gene expressions were dichotomized using a logistic regression spline model [17]. All potential biomarkers including SNPs were separately evaluated for individual association with aggressive disease (D’Amico high-risk vs. low-risk) using unconditional multivariable logistic regression adjusted by age at diagnosis (continuous) and PSA levels at diagnosis (categorical). All biomarkers and SNPs of interest were examined for association with time to BCR or progression using multivariate Cox proportional hazard models adjusted for age, GS, T stage and baseline PSA level. Kaplan–Meier analyses and log-rank tests were used to calculate survival differences. To reduce the likelihood of false discovery, *q*-value for multiple testing was applied in both soluble ICK-related protein and ICK-related genetic variation analysis [18]. The association between genotypes and soluble ICK-related protein levels was analyzed with Spearman correlation. Meta-analysis was performed with the ‘meta’ package in R.

All data were analyzed and visualized with Excel (Microsoft office 365), R software (v3.4.1), PLINK (v1.07), and STATA 14.2 (STATA Corp). All *P* values were two-sided, with values less than 0.05 considered statistically significant.

## Results

### Patient characteristics

We included 1762 individuals with localized PCa in this study. All patients were genotyped, and their clinicopathological features were listed in Table 1. In general, there were 1109 (62.9%) clinical T1, 575 (32.6%) clinical T2, 69 (3.9%) T3–4 PCa. Approximately two thirds (63.0%) of all patients had PSA levels ranging from 4 to 9.9 ng/mL, whereas there were 8.2% of patients at 10–19.9 ng/mL and 3.7% of patients over 20 ng/mL. One third (37.3%) of patients had GS value as 6, whereas 50.3% of patients had GS value as 7 and 7.0% of patients had GS value higher than 7. Among all patients, 100 (5.7%) showed BCR and 39 (2.2%) died.

**Table 1:** Host characteristics of MDACC-Pca, CIT and TCGA cohorts

Variables	MDACC-PCa cohort (*n* %)	Subset of MDACC-Pca cohort		CIT cohort (*n* %)	TCGA cohort (*n* %)
	All	High-risk group^c^	Low-risk group^c^	All	All
Age, mean (SD)	61.60 (7.90)	64.06 (7.60)	64.01 (7.50)	63.21 (6.26)	61.56 (6.78)
Smoking					
Current	149 (8.5)	31 (32.6)	39 (41.1)	NA	NA
Former	770 (43.7)	45 (47.4)	47 (49.5)	NA	NA
Never	826 (46.9)	19 (20.0)	9 (9.5)	NA	NA
Clinical T stage					
T1	1109 (62.9)	34 (35.8)	95 (100.0)	0	0
T2	575 (32.6)	12 (12.6)	0 (0.0)	91 (68.9)	187 (37.9)
T3–T4	69 (3.9)	47 (49.5)	0 (0.0)	40 (30.3)	300 (60.7)
PSA level at diagnosis, ng/mL					
< 4	442 (25.1)	14 (14.7)	33 (34.7)	8 (6.1))	53 (10.7)
4–9.9	1108 (63.0)	54 (56.8)	62 (65.3)	97 (73.4)	275 (55.7)
10–19.9	145 (8.2)	7 (7.4)	0 (0.0)	21 (15.9)	98 (19.8)
≥ 20	65 (3.7)	20 (21.1)	0 (0.0)	3 (2.3)	53 (10.7)
Biopsy-proven GS					
6	657 (37.3)	8 (8.4)	95 (100.0)	66 (50)	45 (9.1)
7	887 (50.3)	27 (28.4)	0 (0.0)	45 (34.1)	246 (49.8)
8	123 (7.0)	35 (36.8)	0 (0.0)	12 (9.1)	64 (13.0)
9	90 (5.1)	23 (24.2)	0 (0.0)	8 (6.1)	135 (27.3)
10	5 (0.3)	2 (2.1)	0 (0.0)	0	4 (0.8)
Dead					
No	1352 (76.7)	89 (93.7)	95 (100.0)	NA	486 (98.4)
Yes	39 (2.2)	6 (6.3)	0 (0.0)	NA	8 (1.6)
Treatment					
Radical prostatectomy	918 (52.10)	53 (55.8)	31 (32.6)	132	NA
Radiotherapy	378 (21.45)	27 (28.4)	14 (14.7)	0	NA
Surveillance/unknown	429 (24.35)	13 (13.7)	47 (49.5)		
Other^b^	37 (2.10)	2 ( 2.1)	3 (3.2)	0	NA
Biochemical recurrence (BCR)^a^					
No	1291 (73.3)	79 (83.2)	95 (100.0)	95 (72.0)	369 (74.7)
Yes	100 (5.7)	16 (16.8)	0 (0.0)	15 (11.4)	57 (11.5)
Total	1762	95	95	132	494

*GS* Gleason Score

^a^Biochemical recurrence (BCR) after local therapy was defined as a single measure of PSA ≥ 0.2 ng/mL after radical prostatectomy, and a PSA rise of 2 ng/mL or more above the nadir PSA in patients who received radiotherapy

^b^Others indicate cryoablation, high-intensity focused ultrasound, transurethral resection

^c^Based on D’Amico criteria for aggressiveness of prostate cancer

A subset of 190 patients was retrieved from the MDACC-PCa cohort, whose serum samples were evaluated for serum ICK-related protein levels. According to D’Amico risk classification, there were 95 patients in the low-risk group and another 95 patients in the high-risk group. There were 34 clinical T1 (35.8%), 12 T2 (12.6%) and 47 T3–4 (49.6%) in the high-risk group, whereas all the patients in the low-risk group had T1 disease. About two thirds (65.3%) of the patients in the low-risk group presented with PSA levels at 4–9.9 ng/mL and in the high-risk group, there were 21.1% of patients over 20 ng/mL, 7.4% at 10–19.9 ng/mL, 56.8% at 4–9.9 ng/mL and 14.7% lower than 4 ng/mL. All patients in the low-risk group had GS = 6, while most patients in the high-risk group had GS > 6 (91.6%). In addition, 84 patients received radical prostatectomy, 41 patients received radiotherapy, 60 patients were under surveillance and 5 patients underwent treatment such as cryoablation.

Demographic information from CIT cohort and TCGA cohort are listed in Table 1 and a schematic diagram is depicted in Figure S1.

### Association of serum ICK-related proteins with PCa aggressiveness, BCR and tumor progression

We evaluated the association between soluble ICK-related proteins and PCa aggressiveness by dichotomizing patients by spline method into high- and low-level groups (Table 2). Among all biomarkers, sTIM3 and sBTLA were associated with D’Amico high-risk disease, and sBTLA had *q*-value lower than 0.15 in multiple comparisons (OR = 2.7, 95% CI 1.3–5.6, *P* = 0.01, *q*-value = 0.14).

**Table 2:** Soluble immune checkpoint factors associated with PCa outcomes

Soluble markers	D'Amico high vs low			BCR			Progression			Cut-off value (pg/mL)
high vs. low levels^a^	Adjusted OR^b^ (95% CI)	*P* value	*q* value	Adjusted HR^c^ (95% CI)	*P* value	*q value*	Adjusted HR^c^ (95% CI)	*P* value	*q value*	
sBTLA	**2.7 (1.3–5.6)**	**0.010**	0.140	4.1 (0.9–17.4)	0.060	0.084	**6.5 (1.9–22.8)**	**0.003**	**0.028**	506.56
sCD27	1.4 (0.8–2.5)	0.250	0.404	1.3 (0.2–6.4)	0.780	0.780	0.4 (0.04–3.6)	0.390	0.455	1846.83
sCD28	0.6 (0.3–1.5)	0.260	0.404	**12.8 (1.6–101.9)**	**0.020**	**0.047**	**7.1 (1.2–42.6)**	**0.030**	**0.047**	2896.50
sCD80	1.1 (0.4–2.6)	0.870	0.870	**5.1 (1.4–19.3)**	**0.020**	**0.047**	**4.2 (1.1–15.9)**	**0.030**	**0.047**	51.24
sCD137	0.8 (0.3–1.8)	0.550	0.592	1.3 (0.2–6.4)	0.780	0.780	2.2 (0.4–13.0)	0.380	0.455	70.62
sCTLA4	5.3 (0.6–46.6)	0.130	0.327	**8.9 (1.3–59.2)**	**0.020**	**0.047**	**8.8 (1.6–48.5)**	**0.010**	**0.028**	89.28
sGITR	0.3 (0.03–3.1)	0.330	0.462	**8.9 (2.1–38.3)**	**0.003**	**0.047**	**7.3 (1.7–30.9)**	**0.007**	**0.028**	124.82
sHVEM	1.4 (0.6–3.4)	0.500	0.583	**7.0 (1.7–29.5)**	**0.008**	**0.047**	**7.0 (1.7–28.9)**	**0.007**	**0.028**	29.00
sIDO	1.9 (0.9–3.7)	0.070	0.245	**6.7 (1.3–35.1)**	**0.020**	**0.047**	**7.9 (1.5–41.9)**	**0.020**	**0.047**	10.51
sLAG3	0.4 (0.1–1.0)	0.060	0.245	1.3 (0.3–5.4)	0.720	0.780	1.1 (0.2–4.7)	0.930	0.930	57.86
sPDCD1	0.3 (0.02–2.3)	0.220	0.404	**5.7 (1.2–27.5)**	**0.030**	**0.053**	**6.4 (1.5–27.2)**	**0.010**	**0.028**	296.17
sPD–L1	1.7 (0.4–7.6)	0.460	0.583	4.8 (0.9–25.7)	0.060	0.084	4.0 (0.8–20.9)	0.100	0.140	17.22
sPD–L2	1.6 (0.9–2.9)	0.140	0.327	**5.5 (1.2–26.2)**	**0.030**	**0.053**	**4.6 (1.1–18.5)**	**0.030**	**0.047**	1420.71
sTIM3	**2.0 (1.0–3.7)**	**0.040**	0.245	1.3 (0.4–4.7)	0.650	0.780	1.5 (0.5–4.5)	0.520	0.560	1413.69

*PCa* prostate cancer, *BCR* biochemical recurrence, *OR* odds ratio, *HR* hazard ratio, *CI* confidence interval

^a^High and low levels were determined after dichotomization using the spline model (Rosenberg et al., 2003). Significant values in bold font

^b^Adjusted for age and baseline PSA level

^c^Adjusted for age, GS, T stage and baseline PSA level

^d^Benjaminiand Hochberg correction method was applied

**Fig. 1:**
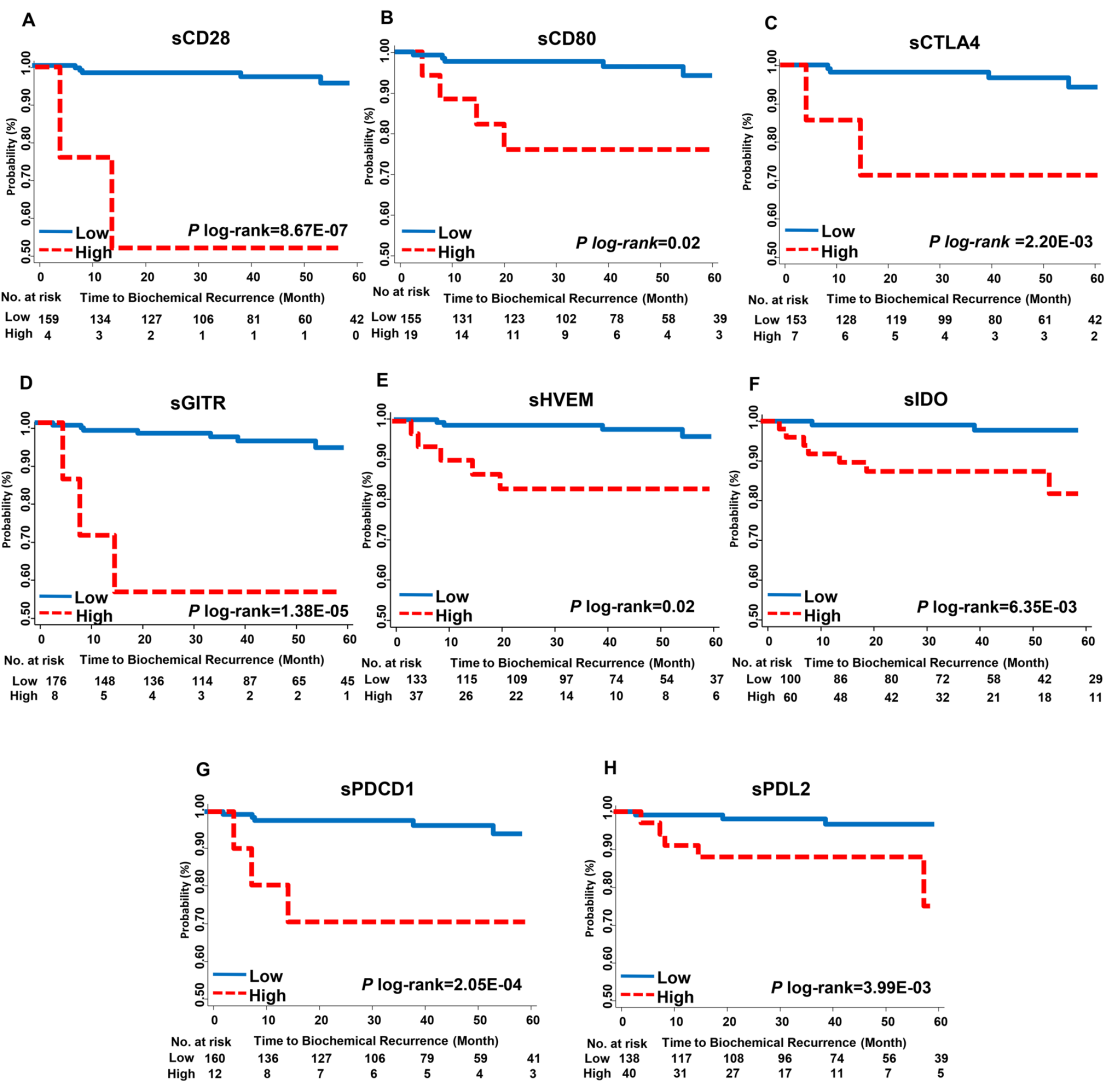
Kaplan Meier analyses of biochemical recurrence (BCR)-free survival by levels of soluble immune checkpoint proteins in localized prostate cancer (PCa) patients. BCR-free survival was estimated according to the levels of **a** sCD28, **b** sCD80, **c** sCTLA4, **d** sHVEM, **e** sIDO, **f** sGITR, **g** sPDCD1, and **h** sPDL2. Patients were dichotomized into high- and low-level groups based on the spline model

We also analyzed the association between 14 ICK-related proteins and BCR in patients with PCa. sCD28, sCTLA4, sHVEM, sIDO, sGITR, sCD80 were significantly correlated with BCR after multiple comparison (Table 2). sGITR demonstrated the most significant association with BCR. The high-sGITR group demonstrated approximately ninefold increased risk of BCR than that of the low-sGITR group (HR, 8.9, 95% CI 2.1–38.3, *P* = 3.4E−03, *q*-value = 0.047). High sGITR was a predictor of poor BCR-free survival in Kaplan–Meier analysis (log-rank*-P* = 1.38E−05; Fig. 1). Similarly, sCD28, sCD80, sCTLA4, sHVEM, sIDO, sPDCD1 and sPD-L2 were also significantly associated with BCR-free survival (log-rank-*P* < 0.05; Fig. 1).

**Fig. 2:**
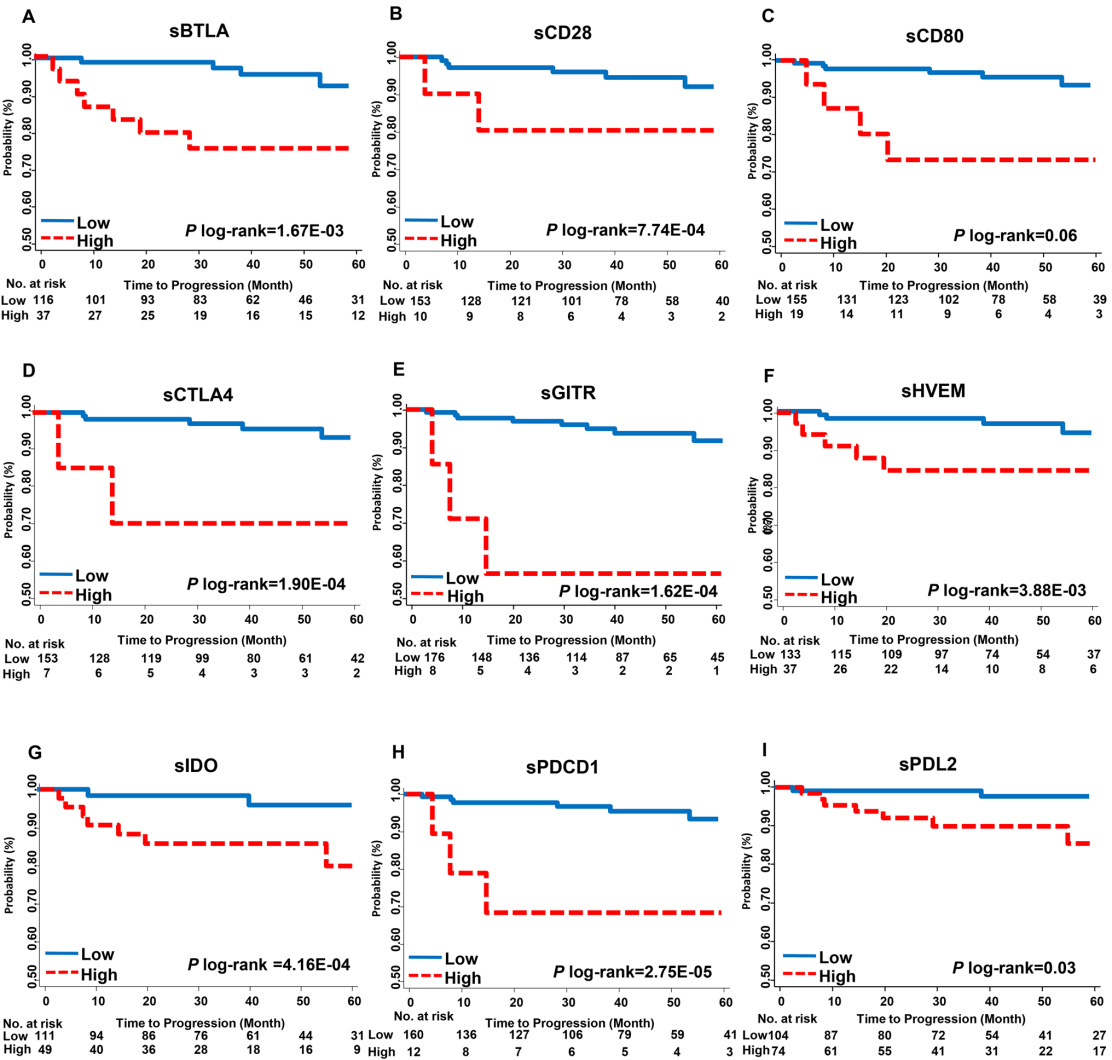
Kaplan Meier analyses of progression-free survival (PFS) by levels of soluble immune checkpoint proteins in patients with localized PCa. Progression-free survival was estimated according to the levels of **a** sBTLA, **b** sCD28, **c** sCD80, **d** sCTLA4, **e** sHVEM, **f** sIDO, **g** sGITR, **h** sPDCD1, and **i** sPDL2. Patients were dichotomized into high- and low-level groups based on the spline model

Further, the associations between serum ICK-related proteins and cancer progression were analyzed. sBTLA, sCD28, sCD80, sCTLA4, sGITR, sHVEM, sIDO, sPDCD1 and sPD-L2 were identified as predictors of PCa progression after multiple comparison (*P* < 0.05). sBTLA is the most significant ICK factor associated with progression (HR, 6.5, 95% CI 1.9–22.8, *P* = 3.3E−03, *q*-value = 0.028). Kaplan–Meier analysis showed the serum factor as a significant predictor of progression-free survival (PFS) (log-rank-*P* = 1.67E−03, Table 2, Fig. 2). sCD28, sCTLA4, sGITR, sHVEM, sIDO, sPDCD1 and sPD-L2 also significantly correlated with PFS (Fig. 2).

### Associations of ICK-related genetic variants with PCa outcomes and functional validation

To investigate the association between ICK-related genetic variations and PCa outcomes, we compared the allele frequencies of 97 SNPs from 19 ICK-related genes for risks of aggressiveness, BCR, and progression. Nine SNPs were associated with risk of aggressive disease. Among those, *LAG3*:rs1997510 conferred the strongest association (OR, 0.34, 95% CI 0.17–0.67, *P* = 0.002, *q*-value = 0.11, Table S1). In addition *D86*:rs17203439 was identified as the most significant SNP correlated with BCR risk among 18 SNPs (HR, 49.2, 95% CI 5.83–414.0, *P* = 3.42E-04, *q*-value = 0.03, Table S2), whereas *CD274*:rs822335 showed the strongest association with PCa progression among 22 SNPs (HR, 1.73, 95% CI 1.31–2.29, *P* = 9.53E−05, *q*-value = 0.009, Table S3). Among all identified SNPs, we observed 8 SNPs significantly associated with both risk of BCR and risk of progression (*P* < 0.05, Table 3).

**Table 3:** Genetic variants of immune checkpoint genes associated with PCa aggressiveness, BCR and progression

Gene	SNP	Location	Model	D'Amico high vs. low		BCR		Progression	
				OR^a^ (95% CI)	*P* value	HR^b^ (95% CI)	*P* value	HR^b^ (95% CI)	*P* value
*BTLA*									
	rs2633562	Intron	DOM	0.61 (0.30–1.24)	0.169	**2.56 (1.40–4.69)**	**0.002**	**2.29 (1.35–3.87)**	**0.002**^c^
*CD80*									
	rs6804441^d^	Intron	REC	–	–	**4.00 (1.72–9.31)**	**0.001**	**3.82 (1.84–7.93)**	**3.24E-04**^c^
	rs12695388^d^	Intron	DOM	0.95 (0.66–1.35)	0.763	**0.61 (0.38–0.98)**	**0.043**	**0.52 (0.36–0.77)**	**0.001**^c^
	rs7628626^d^	3 ‘UTR	REC	–	–	**3.27 (1.47–7.31)**	**0.004**	**2.35 (1.11–4.98)**	**0.025**
	rs491407^d^	Intron	DOM	**2.01 (1.09–3.71)**	**0.026**	0.69 (0.45–1.04)	0.075	0.78 (0.55–1.09)	0.140
*CD274*									
	rs822335	5′ near gene	ADD	1.07 (0.84–1.36)	0.576	**1.78 (1.28–2.47)**	**0.001**	**1.73 (1.31–2.29)**	**9.53E-05**^c^
	rs3780395	Intron	ADD	1.12 (0.89–1.41)	0.335	**0.70 (0.50–0.99)**	**0.044**	**0.63 (0.47–0.85)**	**0.002**^c^
*LAG3*									
	rs12313899	Intron	REC	**0.51 (0.31–0.84)**	**0.008**	0.50 (0.25–1.02)	0.056	0.67 (0.39–1.14)	0.136
	rs12313899	Intron	REC	**0.51 (0.31–0.84)**	**0.008**	0.50 (0.25–1.02)	0.056	0.67 (0.39–1.14)	0.136
*PDCD1LG2*									
	rs6476985	Intron	DOM	1.10 (0.75–1.60)	0.632	**1.74 (1.08–2.81)**	**0.023**	**1.96 (1.32–2.92)**	**0.001**^c^
*TNFSF14*									
	rs2277983	Intron	REC	0.96 (0.65–1.41)	0.836	**0.40 (0.18–0.86)**	**0.020**	**0.54 (0.30–0.96)**	**0.037**

Significant values in bold font

*PCa* prostate cancer, *BCR* biochemical recurrence, *OR* odds ratio, *HR* hazard ratio, *CI* confidence interval, *DOM* dominant, *REC* recessive, *ADD* additive

^a^Adjusted for age and baseline PSA level

^b^Adjusted for age, Gleason score, T stage and baseline PSA level

^c^Significant after Benjaminiand Hochberg correction for multiple testing

^d^Genotype significant associated with soluble CD80 levels

Furthermore, we investigated the relationship between serum biomarkers and corresponding ICK-related genetic variations. Four SNPs located in *CD80* (rs7628626, rs12695388, rs491407 and rs6804441) were significantly correlated with sCD80 level (*P* < 0.05). Among them, rs7628626 demonstrated the strongest association to the sCD80 level (Rho = 0.22, *P* = 0.004, Table S4).

**Fig. 3:**
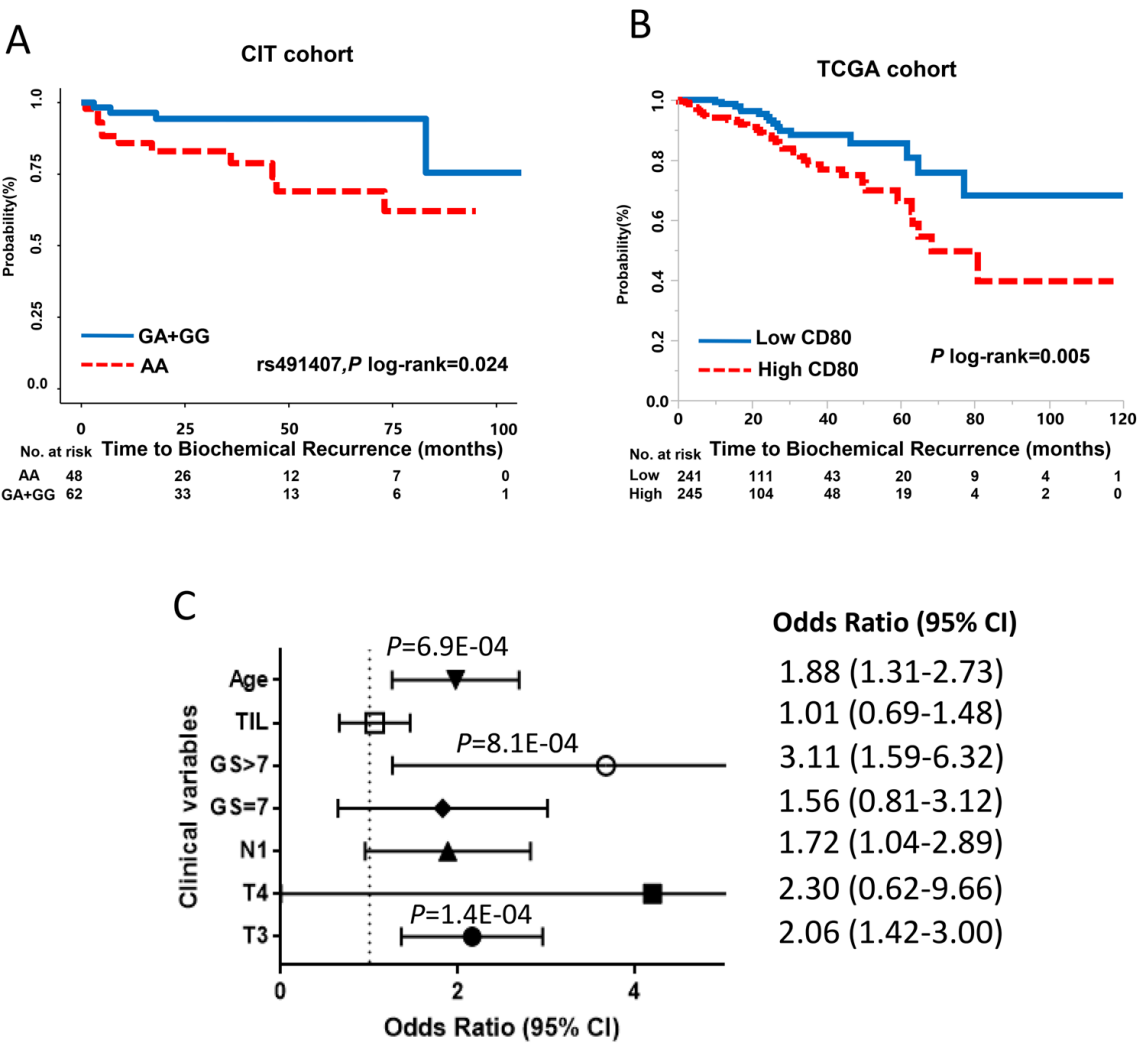
Functional validation of the CD80 genetic variants in CIT cohort and TCGA cohort. **a** BCR-free survival was estimated according to the genotypes of rs491407, patients who carry G allele showed significant better BCR-free survival compare to AA genotype carriers in CIT cohort (log-rank *P* = 0.024). **b** High CD80 expression is significant associated with poor BCR-free survival in TCGA cohort (log-rank *P* = 0.005). **c** CD80 expression is closely associated with age, high GS (> 7), T3 in TCGA cohort

To validate our findings, we turned to the CIT and TCGA cohorts. rs491407 was significantly associated with BCR (HR, 0.23, 95% CI 0.06–0.73, log-rank-*P* = 0.024), which is consistent with MDACC-PCa cohort data (Fig. 3a, Table S5). To examine whether ICK-related genes were altered transcriptionally in PCa, we retrieved 19 ICK-related gene expression data from TCGA. The expression of *CD28, CD274, CTLA4, LAG3, PDCD1LG2, TNFRSF14, TNFRSF18* and *TNFSF18* demonstrated significant differential expression between tumor and normal tissues (*P* < 0.05, Figure S2). Among these genes, *CD80* expression showed significant association with T3 stage, high-GS (> 7) and age. High *CD80* expression correlated with poor BCR-free survival in patients with PCa (Log-rank *P* = 0.005) (Fig. 3b, c). In a meta-analysis of TCGA and CIT cohorts, CD80 was associated with BCR in multi-variate Cox model (HR, 1.85, 95% CI 1.06–3.22, *P* = 0.03) (Table S6).

## Discussion

In this study, we found that serum ICK-related proteins and genetic variations in ICK-related genes were associated with outcomes of localized PCa. Specifically, high levels of sBTLA and sTIM3 correlated with the risk of aggressive PCa. sCD28, sCD80, sCTLA4, sHVEM, sIDO, sGITR, sPDCD1, and sPDL2 were significantly correlated with both risks of BCR and PCa progression, whereas sBTLA was also identified as a predictor of PCa progression. We demonstrated that genetic variations of ICK-related genes were significantly associated with PCa outcomes. A total of 8 SNPs were associated with risks of BCR and progression. Interestingly, the genotypes of four CD80 variants were significantly correlated with sCD80 level. rs491407 was further validated in an independent cohort. *CD80* expression was also identified as a prognostic biomarker for BCR-free survival in the combined analysis of CIT and TCGA cohort. To the best of our knowledge, this is the first study to demonstrate that serum proteins and SNPs of ICK-related genes are predictive of PCa outcomes, and the correlation between sCD80 level and *CD80* genotypes may implicate a potential functional mechanism.

As an important co-signaling molecule expressed on the surface of antigen-presenting cells, CD80 binds to CD28 or CTLA4 to activate T cell co-stimulation or initiate T cell coinhibition, respectively [19]. One previous study has reported that B7 family members in tumors, specifically B7-H3 and B7x, are associated with disease spread and poor survival in PCa [20], but few studies have focused on B7-1 (CD80). Eugene et al. report that ectopic CD80 expression in a murine PCa cell line pTC1 transplanted to an in vivo mouse model could elicit immune elimination of tumor cells [21]. However, our study demonstrated consistent correlations between the cellular and circulating forms of CD80. It has been reported that human sCD80 is generated by alternative splicing of the gene and that a recombinant form inhibits T cell activation and proliferation in vitro [22]. Thus, sCD80 may bind to CTLA4 and initiate T cell co-inhibition, which could lead to immune escape of tumor cells and elevated risk of recurrence [23]. In addition, sCD80 may compete with membranous CD80 on APCs to modulate its costimulatory effect on T cells. To date, no evidence of sCD80′s therapeutic effect in human cell model has been reported, which requires more research.

We identified four SNPs located in *CD80* loci that were associated with sCD80 level as well as cancer outcomes. rs491407 was further validated in another independent cohort as a predictor of BCR. rs7628626, located in the 3′UTR of *CD80* gene, was associated with BCR and progression. This is consistent with a previous finding that genotype of rs7628626, targeted by miR-21-3p, is correlated with regional lymph node metastasis and tumor progression in colon cancer [24]. Interestingly, miR-21 could also promote tumor invasion and is associated with increased BCR in prostate cancer [25], which may explain our finding that *CD80* expression predicts BCR-free survival in a combined analysis of CIT and TCGA cohort. Therefore, we propose that the mechanism between the genotype of rs7628626 and BCR and progression in localized PCa may involve miR-21 that can functionally regulate CD80 expression leading to differential serum and membranous CD80 levels.

BTLA is an inhibitory ICK interacting with HVEM and LIGHT in the surface of antigen-presenting cells [26]. In this study, we identified sBLTA as a predictor of aggressive disease and a high risk of progression in localized PCa for the first time. In addition, sHVEM was identified as a predictor of the high risk of BCR and progression. HVEM-BTLA binding resulted in an inhibitory effect on T cell activation and proliferation, which lead to impeded anti-tumor immunity [27], which may explain why both sHVEM and sBTLA predicting poor outcomes in PCa. Further, we identified rs2633562 and rs1982809 located in *BTLA* gene region, were associated with increased risk of BCR and progression. However, we failed to identify the correlation between sBTLA and the genotypes of this variant. Thus, our findings require further investigation to elucidate the underlying mechanism of association.

GITR is a co-stimulatory TNF receptor superfamily member, which affords the potential to expand CD8+ T cell population [28]. In our study, sGITR is associated with increased risk of BCR and progression, which is not consistent with its co-stimulatory role in T cell activation. The inconsistency may derive from the high GITR expression in Treg cells [29], which were highly infiltrated in prostate tissues [30]. Any immune suppression induced by Treg cell may contribute to BCR and progression. However, this will require further research to decipher the underlying mechanisms.

We also identified other serum ICK-related proteins associated with outcomes of PCa. For example, sPDL2 was correlated with increased risk of BCR, and sTIM3 was predictive of the high risk of aggressive PCa. Both PDL2 and Tim3 are reported as potential targets for cancer immunotherapy [31, 32].

Our study has distinct advantages in the multi-phase study design within a large and high-quality cohort, multiplex serum profiling of serum ICK-related proteins and further genotypic analysis of ICK-related genes, analysis of genotype–phenotype correlation, and validation which brings biological validity to some of our findings. However, we also acknowledge several limitations. First, our study was conducted in a single hospital-based cohort. Additional validation in another independent cohort is warranted. Second, cellular ICK gene expression in peripheral blood mononuclear cells (PBMCs) was not evaluated in this study due to the availability and viability of PBMC samples. The correlation between soluble and cellular ICK-related proteins in peripheral blood is unknown. Nevertheless, we analyzed the expression of selected ICK-related genes based on TCGA data to evaluate the ICK phenotypes in tumors. Third, we only assessed serum ICK protein levels in a small subset of patients with PCa from the cohort. The relatively small sample size and outcome events may restrict the study power. Fourth, as the functional implications in this study are conducted in silico, further validation in independent cohorts or in the laboratory is warranted.

In conclusion, we identified a panel of serum ICK-related proteins and genetic variations that were associated with outcomes of localized PCa. Significant genotype–phenotype associations were identified between sCD80 and genetic variants in *CD80*. Further validations indicated that rs491407 and *CD80* expression were correlated with BCR. These findings point to the potential prognostic values of serum ICK-related proteins and genetic variants, which may also provide potential therapeutic targets for immunotherapy of localized PCa.

## Electronic supplementary material

Below is the link to the electronic supplementary material.

Supplementary file 1 (DOCX 1019 kb)

## Data Availability

The datasets used and analyzed during the current study are available from the corresponding author on reasonable request.
