# Supplementary material for: Immune checkpoint-related serum proteins and genetic variants predict outcomes of localized prostate cancer, a cohort study
Source: Cancer Immunol Immunother. 2020 Sep 9;70(3):701–12. doi: 10.1007/s00262-020-02718-1 (PMC7907032; doi:10.1007/s00262-020-02718-1)
Supplement: Supplementary file 1 — (DOCX 1019 kb) [file 262_2020_2718_MOESM1_ESM.docx]

**Supplemental Materials**

Immune checkpoint serum proteins and genetic variants predict outcomes of localized prostate cancer

Qinchuan Wang, Hao Yu, Shu-hong Lin, Huakang Tu, Dong Liang, Yuanqing Ye, David. W Chang, Xifeng Wu

**Supplemental Tables**

| **Supplemental Table S1. Genetic variants of immune checkpoint genes associate with PCa aggressiveness.** | | | | | | | |
| --- | --- | --- | --- | --- | --- | --- | --- |
| **Gene** | **SNP** | **Location** | **Model** | **D'Amico High** | **D'Amico Low** | **OR* (95% CI)** | ***P* value** |
| *LAG3* |  |  |  |  |  |  |  |
|  | rs1997510 | Intron | REC | 166\138\26 | 294\230\74 | 0.34(0.17-0.67) | 0.002 |
|  | rs12313899 | Intron | REC | 130\156\44 | 226\265\107 | 0.51(0.31-0.84) | 0.008 |
| *PDCD1LG2* | |  |  |  |  |  |  |
|  | rs7025291 | Intron | ADD | 120\159\51 | 180\307\111 | 0.69(0.54-0.87) | 0.002 |
| *CD80* |  |  |  |  |  |  |  |
|  | rs491407 | Intron | REC | 190\118\22 | 332\233\33 | 2.01(1.09-3.71) | 0.026 |
| *LGALS9* |  |  |  |  |  |  |  |
|  | rs3763959 | Upstream near gene | ADD | 94\163\73 | 207\285\104 | 1.32(1.05-1.66) | 0.019 |
|  | rs4794975 | Intron | ADD | 178\130\8 | 286\241\38 | 0.75(0.57-1.00) | 0.047 |
| *CD27* |  |  |  |  |  |  |  |
|  | rs3136550 | Intron | DOM | 158\147\25 | 313\230\55 | 1.41(1.02-1.94) | 0.039 |
| *CD70* |  |  |  |  |  |  |  |
|  | rs1862511 | Synonymous codon | REC | 160\132\37 | 314\245\33 | 1.94(1.08-3.49) | 0.027 |
| *CD86* |  |  |  |  |  |  |  |
|  | rs9831894 | Intron | DOM | 103\168\59 | 222\276\100 | 1.45(1.03-2.05) | 0.035 |

Abbreviations: PCa, prostate cancer; OR, odds ratio; CI, confidence interval, DOM, dominant; REC, recessive;

ADD, additive.

* Adjusted with age, GS, T stage and baseline PSA level.

| **Supplemental Table S2. Genetic variants of immune checkpoint genes associated with BCR risk.** | | | | | | | |
| --- | --- | --- | --- | --- | --- | --- | --- |
| **Gene** | **SNP** | **Location** | **Model** | **BCR** | **No BCR** | **HR* (95% CI)** | ***P* value** |
| *CD86* |  |  |  |  |  |  |  |
|  | rs17203439 | Intron | REC | 85\14\1 | 1140\150\1 | 49.2(5.83-414.0) | 3.42E-04^‡^ |
|  | rs2254911 | Intron | REC | 84\15\1 | 1106\180\2 | 27.6(3.44-221.9) | 0.002^‡^ |
| *CD80* |  |  |  |  |  |  |  |
|  | rs6804441 | Intron | REC | 63\28\7 | 863\394\33 | 4.00(1.72-9.31) | 0.001^‡^ |
|  | rs7628626 | 3’UTR | REC | 67\26\7 | 851\400\40 | 3.27(1.47-7.31) | 0.004 |
|  | rs12695388 | Intron | DOM | 34\43\23 | 311\667\312 | 0.61(0.38-0.98) | 0.043 |
|  | rs1880661 | Upstream near gene | REC | 30\58\12 | 400\645\246 | 0.48(0.24-0.98) | 0.043 |
| *CD274* |  |  |  |  |  |  |  |
|  | rs822335 | Promoter region | ADD | 35\51\14 | 558\587\146 | 1.78(1.28-2.47) | 0.001^‡^ |
|  | rs3780395 | Intron | REC | 34\56\10 | 458\621\212 | 0.40(0.17-0.94) | 0.036 |
| *TNFRSF18* |  |  |  |  |  |  |  |
|  | rs3819001 | 3‘ UTR | REC | 89\10\1 | 1159\131\1 | 19.4(2.35-159.7) | 0.006 |
| *TNFSF14* |  |  |  |  |  |  |  |
|  | rs2277983 | Intron | ADD | 38\51\11 | 380\621\290 | 0.67(0.48-0.93) | 0.018 |
|  | rs11569562 | Intron | ADD | 37\48\15 | 355\623\312 | 0.72(0.53-1.00) | 0.047 |
| *BTLA* |  |  |  |  |  |  |  |
|  | rs2633562 | Intron | DOM | 87\13\0 | 1201\87\3 | 2.56(1.40-4.69) | 0.002 |
|  | rs1982809 | 3‘ UTR | ADD | 54\36\10 | 782\442\67 | 1.41(1.01-1.99) | 0.047 |
|  | rs2633578 | Upstream near gene | DOM | 79\21\0 | 1089\195\7 | 1.67(1.00-2.80) | 0.050 |
| *CD70* |  |  |  |  |  |  |  |
|  | rs16994592 | Intron | REC | 84\13\3 | 1040\241\10 | 4.47(1.37-14.5) | 0.013 |
|  | rs2910434 | Downstream near gene | REC | 81\16\3 | 1020\256\15 | 3.92(1.21-12.7) | 0.023 |
| *PDCD1LG2* |  |  |  |  |  |  |  |
|  | rs6476985 | Intron | DOM | 69\31\0 | 1018\255\18 | 1.74(1.08-2.81) | 0.023 |
| Abbreviations: BCR, biochemical recurrence; HR, hazard ratio; CI, confidence interval; UTR, untranslated region; DOM, dominant; REC, recessive; ADD, additive.  * Adjusted with age, GS, T stage and baseline PSA level.  ‡ Significant after Benjaminiand Hochberg correction for multiple testing. | | | | | | | |

**Supplemental Table S3. Genetic variants of immune checkpoint genes associated with PCa progression.**

| **Gene** | **SNP** | **Location** | **Model** | **Progression** | **No Progression** | **HR* (95% CI)** | ***P* value** |
| --- | --- | --- | --- | --- | --- | --- | --- |
| *CD274* |  |  |  |  |  |  |  |
|  | rs822335 | Upstream near gene | ADD | 48\67\20 | 520\545\133 | 1.73(1.31-2.29) | 9.53E-05^‡^ |
|  | rs3780395 | Intron | ADD | 52\70\13 | 420\578\200 | 0.63(0.47-0.85) | 0.002^‡^ |
|  | rs822349 | Upstream near gene | ADD | 27\75\33 | 324\581\292 | 1.45(1.10-1.90) | 0.008 |
|  | rs4742100 | Intron | REC | 73\48\14 | 716\419\63 | 2.27(1.24-4.16) | 0.008 |
| *PDCD1LG2* |  |  |  |  |  |  |  |
|  | rs6476985 | Intron | DOM | 91\44\0 | 950\231\17 | 1.96(1.32-2.92) | 0.001^‡^ |
| *CD80* |  |  |  |  |  |  |  |
|  | rs6804441 | Intron | REC | 83\41\9 | 803\365\29 | 3.82(1.84-7.93) | 3.24E-04^‡^ |
|  | rs12695388 | Intron | DOM | 50\55\30 | 283\622\292 | 0.52(0.36-0.77) | 0.001^‡^ |
|  | rs7628626 | 3’UTR | REC | 95\32\8 | 787\376\35 | 2.35(1.11-4.98) | 0.025 |
| *BTLA* |  |  |  |  |  |  |  |
|  | rs2633562 | Intron | DOM | 118\17\0 | 1116\80\2 | 2.29(1.35-3.87) | 0.002^‡^ |
|  | rs1982809 | 3’UTR | REC | 80\41\14 | 720\416\62 | 2.07(1.13-3.80) | 0.019 |
| *CD27* |  |  |  |  |  |  |  |
|  | rs11064196 | Intron | DOM | 39\74\22 | 492\529\177 | 1.69(1.13-2.53) | 0.011 |
| *CD28* |  |  |  |  |  |  |  |
|  | rs41272649 | Intron | DOM | 127\8\0 | 1169\27\0 | 2.75(1.20-6.33) | 0.017 |
| *CD70* |  |  |  |  |  |  |  |
|  | rs16994592 | Intron | REC | 113\18\4 | 966\223\9 | 3.75(1.34-10.5) | 0.012 |
|  | rs2910434 | Downstream  near gene | REC | 109\22\4 | 948\236\14 | 3.22(1.15-8.96) | 0.025 |
| *CD86* |  |  |  |  |  |  |  |
|  | rs17203439 | Intron | REC | 114\20\1 | 1058\140\0 | 25.3(3.07-209.8) | 0.003 |
|  | rs2254911 | Intron | REC | 113\21\1 | 1026\168\1 | 14.2(1.83-110.9) | 0.011 |
|  | rs2715274 | Intron | REC | 86\40\9 | 771\375\52 | 2.13(1.07-4.24) | 0.032 |
|  | rs1129055 | Missense | REC | 72\60\3 | 621\478\99 | 0.30(0.09-0.94) | 0.039 |
| *LAG3* |  |  |  |  |  |  |  |
|  | rs12313899 | Intron | DOM | 37\81\17 | 443\534\221 | 1.74(1.13-2.66) | 0.011 |
| *TNFSF14* |  |  |  |  |  |  |  |
|  | rs2277983 | Intron | REC | 46\72\17 | 354\581\263 | 0.54(0.30-0.96) | 0.037 |
| *TNFSF9* |  |  |  |  |  |  |  |
|  | rs12151125 | Intron | DOM | 58\57\20 | 463\558\177 | 0.68(0.47-1.00) | 0.047 |

Abbreviations: PCa, prostate cancer; OR, odds ratio; CI, confidence interval, DOM, dominant; REC, recessive;

ADD, additive.

* Adjusted with age, GS, T stage and baseline PSA level.

‡ Significant after Benjaminiand Hochberg correction for multiple testing

| **Supplemental Table S4: Association between soluble immune checkpoint biomarkers and immune genetic variants** | | | | | |
| --- | --- | --- | --- | --- | --- |
| **MDACC-PCa cohort** | | | | | |
| **SNP** | **Model** | **Biomarkers** | **N** | **Rho** | ***P* value** |
| rs7628626 | REC | sCD80 | 169 | 0.221 | 0.004 |
| rs12695388 | DOM | sCD80 | 169 | 0.220 | 0.004 |
| rs491407 | DOM | sCD80 | 169 | 0.200 | 0.009 |
| rs6804441 | REC | sCD80 | 168 | -0.178 | 0.021 |

| **Supplemental Table S5: Validation of genetic variants in CD80 in CIT cohort** | | | |
| --- | --- | --- | --- |
| **SNP** | **Model** | ***HR(95%CI)** | ***P* value** |
| rs7628626 | REC | 0.54(0.18-1.74) | 0.279 |
| rs12695388 | DOM | 2.33(0.69-10.75) | 0.213 |
| rs491407 | DOM | 0.23(0.06-0.73) | 0.019 |
| rs6804441 | REC | 2.89(0.80-9.53) | 0.086 |

* Adjusted with age, GS, T stage and baseline PSA level.

| **Supplemental Table S6: Multi-variable COX proportional hazard model based on TCGA dataset** | | | | | | |
| --- | --- | --- | --- | --- | --- | --- |
|  | TCGA cohort |  | CIT cohort |  | Meta analysis |  |
|  | *Adjusted HR (95%CI) | *P* value | *Adjusted HR (95%CI) |  | *Adjusted HR (95%CI) | *P* value |
| CD80 |  |  |  |  |  |  |
| Low | Reference |  | Reference |  | Reference |  |
| High | 1.80 (0.97-3.51) | 0.06 | 2.02 (0.65-6.27) | 0.22 | 1.85 (1.06-3.22) | **0.03** |
| *Adjusted with clinical T stage, Gleason score, pre-operative PSA level and age. | | | |  |  |  |

**Supplemental Figures**


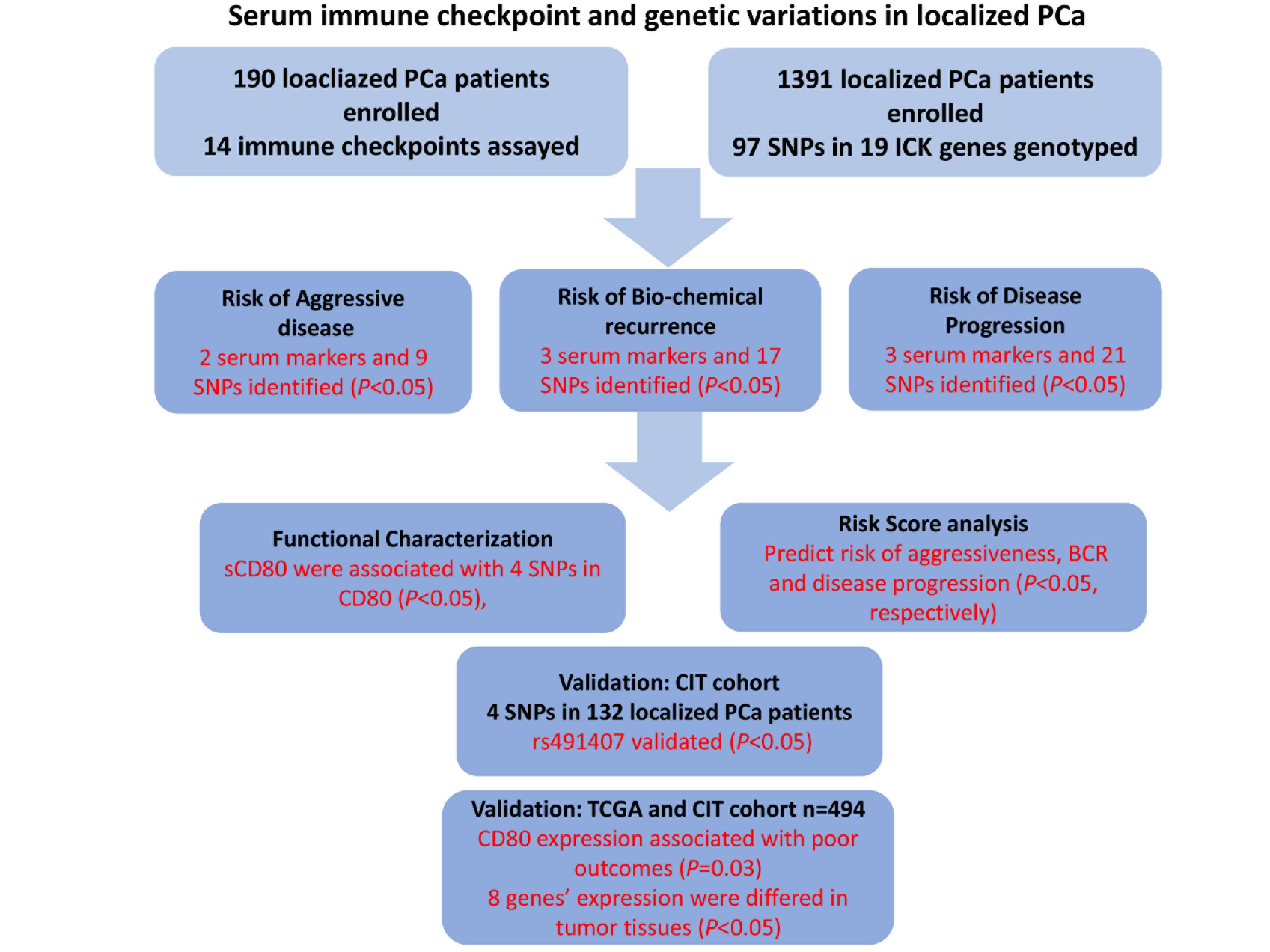


**Supplemental Figure S1. Study design and workflow.**


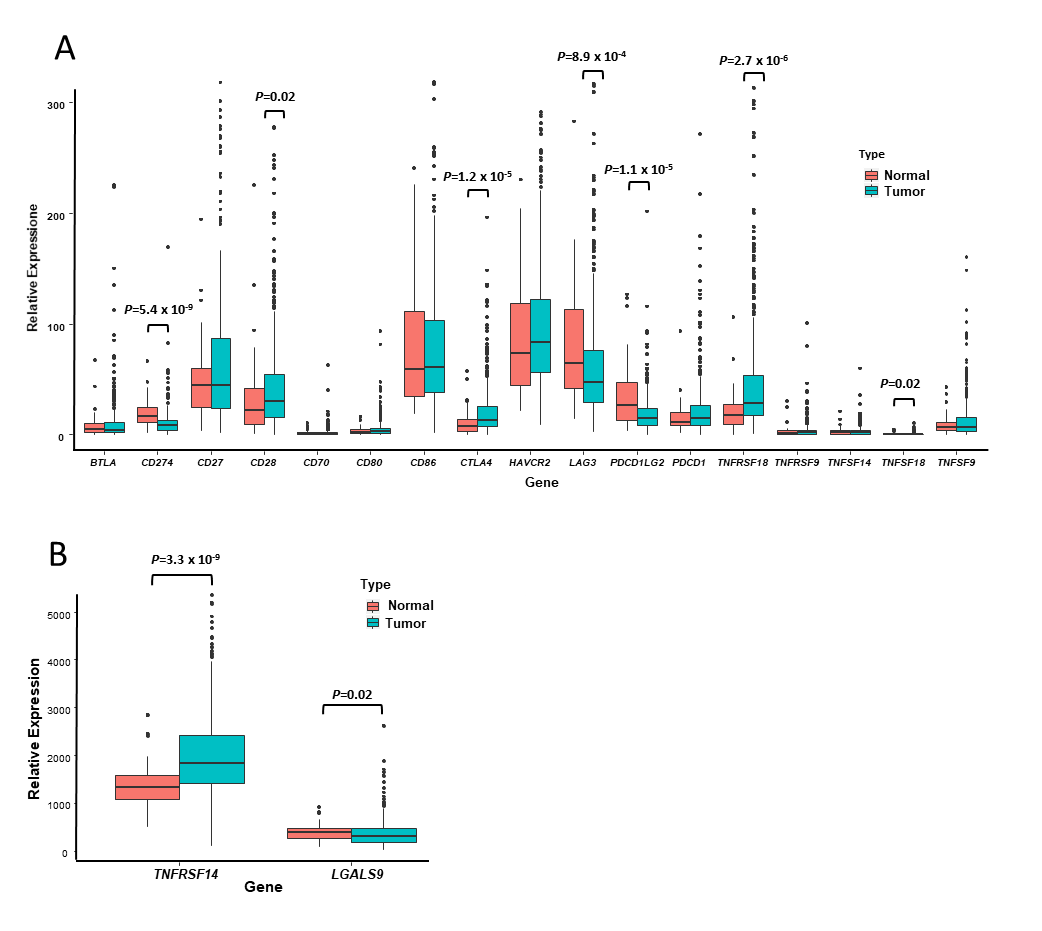


**Supplemental Figure S2. Tumor/normal tissue expression of immune checkpoint genes in The Cancer Genome Atlas.**

**(TCGA) database**

RNA sequencing data were retrieved from TCGA database for prostate cancer and analyzed for 497 tumor tissues and 52 normal tissues. Expression of *CD274*, *CD28*, *CTLA4*, *LAG3*, *PDCD1LG2*, *TNFRSF18* and *TNFSF18* (panel A) and *TNFRSF14* and *LGALS9* (panel B) showed differential expression between tumor and normal tissues (*P*<0.05).
